# An Improved Elk Herd Optimization Algorithm for Maximum Power Point Tracking in Photovoltaic Systems Under Partial Shading Conditions

**DOI:** 10.3390/biomimetics10080533

**Published:** 2025-08-13

**Authors:** Gang Zheng, Wenchang Wei, Heming Jia, Yiqi Liu, Jiankai Lin

**Affiliations:** 1 College of Computer and Control Engineering, Northeast Forestry University, Harbin 150040, China; weiwenchang@nefu.edu.cn (W.W.); ee_617@nefu.edu.cn (Y.L.); 2024112776@nefu.edu.cn (J.L.); 2 School of Information Engineering, Sanming University, Sanming 365004, China

**Keywords:** improved elk herd optimization, maximum power point tracking, photovoltaic system, partial shading condition

## Abstract

In partial shading conditions (PSCs), the power–voltage characteristics of photovoltaic systems exhibit multiple peaks, causing traditional maximum power point tracking (MPPT) algorithms to easily become trapped in local optima and fail to achieve global maximum power point tracking, thereby reducing energy conversion efficiency. Effectively and rapidly locating the global maximum power under complex environmental conditions has become crucial for enhancing MPPT performance in photovoltaic systems. This paper therefore proposes an improved elk herd optimization (IEHO) algorithm to achieve the rapid tracking of the global maximum power point under various weather conditions. The algorithm proposes a position update mechanism guided by the predation risk probability to direct elk herd migration and introduces the triangle walk strategy, thereby enhancing the algorithm’s capability to avoid local optima. Furthermore, IEHO employs a memory-guided redirection strategy to skip redundant calculations of historical duty cycles, significantly improving the convergence speed of MPPT. To validate the algorithm’s performance advantages, the proposed IEHO method is compared with other recognized meta-heuristic algorithms under various weather conditions. The experimental results demonstrate that, across all tested conditions, the proposed IEHO method achieves an average tracking efficiency of 99.99% and an average tracking time of 0.3886 s, outperforming other comparative algorithms.

## 1. Introduction

Currently, the global energy structure remains predominantly reliant on fossil fuels, with coal, oil, and natural gas accounting for the majority of energy consumption in many countries. While conventional power generation technologies have achieved relative stability, they face multiple challenges including finite reserves, environmental pollution, and low efficiency. Transitioning from fossil fuel-based energy systems to low-carbon alternatives and ultimately establishing a sustainable energy era centered on renewable sources has become a fundamental trend in global energy transformation [1,2]. As a critical component of renewable energy systems, photovoltaic (PV) systems are playing an increasingly vital role in the global energy mix due to their clean and sustainable characteristics. With the widespread adoption of PV systems in renewable energy applications, improving power generation efficiency and reducing costs have become focal priorities for the photovoltaic industry. The core issue for improving power generation efficiency lies in accurately and rapidly achieving maximum power point tracking (MPPT) for PV systems. Consequently, MPPT technology has emerged as a pivotal means to enhance energy conversion efficiency. However, the energy conversion efficiency of PV systems is significantly influenced by environmental conditions such as irradiance, temperature, and shading effects, with power losses caused by partial shading conditions (PSCs) being particularly prominent. In PSCs, the power–voltage (P-V) characteristics of PV arrays exhibit multi-peak characteristics, rendering conventional MPPT algorithms ineffective in accurately identifying the global maximum power point (GMPP), resulting in substantial efficiency reduction [3,4,5,6,7].

Under uniform irradiation conditions, the P-V curve exhibits a single-peak characteristic. Conventional MPPT control methods, including constant voltage tracking (CVT) [8,9], perturbation and observation (P&O) [10], hill climbing (HC) [11], and incremental conductance (INC) [12], can effectively track the maximum power point. However, when PV arrays experience partial shading, irradiation differences between cell strings induce current mismatch, triggering the conduction of bypass diodes. This phenomenon splits the P-V curve into multiple local maximum power points (LMPPs) and a single global maximum power point (GMPP). In such scenarios, conventional algorithms with fixed-step strategies become prone to becoming trapped in local optimum, leading to power loss and steady-state oscillations. More critically, dynamic shading conditions further exacerbate MPPT challenges [13]. These situations require the algorithm to possess rapid response capability and dynamic adaptability, as GMPP locations vary in real-time with irradiation distribution changes.

In recent years, artificial intelligence (AI) algorithms have been progressively applied across various fields due to their exceptional data processing speed, precision, and inherent data training capabilities. Researchers have extended their application to MPPT control systems, including artificial neural networks (ANNs) [14], convolutional neural networks (CNNs) [15], and fuzzy logic control (FLC) [16]. The integration of AI methods has further enhanced the adaptability of MPPT algorithms in complex environments. However, the substantial computational resources and large datasets required for inference and training pose higher demands on hardware conditions in practical applications.

Swarm intelligence optimization algorithms are a class of optimization techniques inspired by collective behaviors observed in natural populations, such as bird flocks, fish schools, and ant colonies. These algorithms resolve complex optimization problems by simulating collaborative mechanisms of biological groups. Swarm intelligence optimization algorithms demonstrate significant advantages in addressing a wide range of optimization problems and complex tasks while exhibiting robust performance in multimodal optimization. Consequently, researchers have extensively explored their applications in MPPT. Commonly employed swarm intelligence optimization algorithms for MPPT include particle swarm optimization (PSO) [17,18,19], gref wolf optimization (GWO) [20,21], cuckoo search (CS) [22], a genetic algorithm (GA) [23,24], and differential evolution (DE) [25]. Javed et al. [26] proposed a simplified yet fully adaptive PSO algorithm, utilizing two adaptive parameters to independently regulate particle velocity and search space variation. This design enables precise maximum power point localization without compromising tracking speed. To address partial shading conditions, Sangrody et al. [27] developed an enhanced PSO-based MPPT technique that estimates the convex region of the power–voltage (P-V) curve using dual voltage boundaries. The proposed algorithm achieves rapid convergence and locates the GMPP under small fluctuations. Laxman et al. [28] integrated GWO with adaptive fuzzy logic control for MPPT applications. The GWO was utilized to optimize membership functions, thereby generating the optimal duty cycle at the maximum power point. This hybrid algorithm could track the GMPP under all shading conditions while reducing oscillations and improving tracking efficiency. Águila-León et al. [22], Wei et al. [23], and Yadav et al. [24] have combined two swarm intelligence algorithms for PV MPPT technology. By leveraging the complementary strengths of algorithm integration, they addressed issues such as local convergence and power oscillations inherent in single-algorithm approaches, thereby effectively enhancing the energy conversion efficiency of photovoltaic arrays.

Jia et al. [29] developed an MPPT method integrating Harris hawk optimization (HHO) with a variable-step incremental conductance method. The variable-step incremental conductance method was switched to local exploration, enhancing the HHO algorithm’s ability to escape local optima while reducing power oscillations. Jamaludin et al. [30] proposed a salp swarm algorithm (SSA) inspired by the swarm behavior of salp predation for MPPT. The SSA successfully identified the MPP and improved PV cell utilization efficiency. Kong et al. [31] proposed an improved snake optimizer (ISO) algorithm for MPPT control. By introducing chaotic mapping to increase population diversity and employing the levy flight strategy combined with dynamic exploitation probability, the algorithm enhanced global search capability and avoided local optima. Refaat et al. [32] developed a novel method using the horse herd optimization (HHO) algorithm to extract maximum power from PV systems under varying weather conditions. This approach maintained high maximum power tracking accuracy and shorter tracking times across all-weather scenarios. Naser et al. [33] presented a modified coot optimization algorithm. With only a single tuning parameter and by skipping unnecessary search spaces, the algorithm improved the convergence speed of MPPT. Sushmi et al. [34] introduced a beluga whale optimization (BWO)-based MPPT algorithm. The levy flight function and whale fall process strengthened global exploration capability, achieving faster and smoother convergence while effectively tracking the GMPP. Mai et al. [35] proposed a dung beetle optimization (DBO)-based MPPT technique. This method effectively addressed random power oscillations, low tracking efficiency, and local optimum entrapment in PSCs. Li et al. [36] investigated a bio-inspired sand cat swarm optimization (SCSO)-based MPPT algorithm. Under partial shading and dynamic environmental conditions, this approach achieved the rapid tracking of higher output power within shorter timeframes. Abdelmalek et al. [37] proposed the zebra optimization algorithm (ZOA) as a novel method to monitor the GMPP of PV systems. The experimental results validated the algorithm’s balanced global exploration and local exploitation capabilities.

In recent years, although swarm intelligence optimization algorithms have demonstrated potential in multimodal optimization problems, they still face critical challenges in convergence speed, robustness, and computational efficiency under complex partial shading conditions. The elk herd optimization (EHO) [38] algorithm, modeled after the collective behavior of elk herds, exhibits strong global search capability without requiring complex parameter tuning. However, the EHO suffers from low convergence accuracy, slow tracking speed, and susceptibility to local optima.

The main contributions of this paper are summarized as follows:

This study focuses on improving MPPT technology under complex PSCs. An enhanced MPPT technique based on the Improved Elk Herd Optimizer (IEHO) algorithm is proposed to optimize the performance of PV systems under various weather conditions.

In the predation risk avoidance migration mechanism, the predation risk probability is introduced to simulate the migration process of elk herds toward safer environments, thereby enhancing the algorithm’s global exploration capability. By incorporating the triangle walk strategy and combining the memory-guided redirection strategy, the algorithm’s ability to evade local optima is further strengthened. These modifications significantly improve convergence speed, enabling the IEHO algorithm to rapidly and accurately track the GMPP.

The proposed IEHO algorithm was compared with other recognized metaheuristic MPPT techniques, including EHO, PSO, GWO, SCSO, the SSA, and RIME [39], to validate the effectiveness of the proposed method. The experimental results demonstrated that the proposed algorithm exhibits significant advantages in core performance metrics, such as tracking efficiency and convergence speed, under both uniform irradiation and all PSCs.

The remainder of this paper is organized as follows: Section 2 provides a comprehensive discussion on the modeling of PV systems under partial shading conditions. Section 3 outlines the standard EHO methodology. Section 4 details the IEHO algorithm. Section 5 presents the simulation results validating the proposed method under uniform irradiation and four PSCs, with performance comparisons against other metaheuristic MPPT algorithms using various evaluation metrics. Section 6 concludes the paper.

## 2. Analysis of Principles and Characteristics of PV Systems

The energy conversion efficiency of PV power generation systems is directly determined by the performance of their MPPT controllers, whose primary task involves the real-time adjustment of the PV array’s operating point to ensure global maximum power capture under complex environmental conditions. PV cells, as the energy source of the system, demonstrate output characteristics such as the nonlinear relationships among voltage, current, and power, which form the fundamental basis for the design of MPPT algorithms. Due to dynamic influences from environmental factors including irradiance intensity, temperature, and partial shading, the P-V curve of PV cells may exhibit multi-peak characteristics. This phenomenon causes conventional MPPT algorithms to become trapped in local extrema, resulting in significant power losses. Therefore, an in-depth investigation of the mathematical models and output characteristics of PV cells constitutes a key theoretical prerequisite for enhancing system power generation efficiency.

### 2.1. PV Cell Mathematical Model

**Figure 1:**
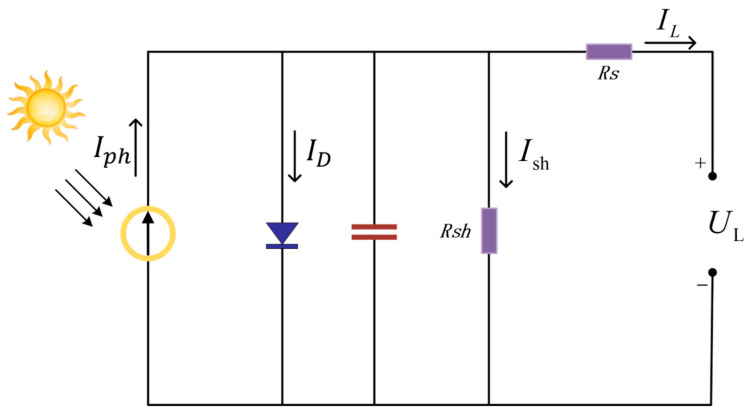
Equivalent circuit model of PV cell.

PV cells convert light energy into electrical energy based on the PV effect. The core physical process involves light illumination exciting electron–hole pairs at the semiconductor PN junction, which are subsequently separated under the influence of the built-in electric field to generate photocurrent $I_{ph}$. The single-diode equivalent circuit model (Figure 1) is widely employed to describe the output characteristics of PV cells. The mathematical model for the current–voltage (I–V) characteristics of a PV module is defined by the following set of equations [40].

(1) $$I_{L}=I_{ph}-I_{D}-I_{sh}$$

where ${\;I}_{L\;}$ represents the output current, $I_{ph}$ denotes the current source, ${\;I}_{sh}$ is the current through the equivalent parallel resistor, and ${\;I}_{L}\;$ is calculated by the following formula:

(2) $$\begin{array}{c} I_{L}=I_{ph}-I_{D}\times \left[exp\left(\frac{q\left(U_{L}+I_{L}{*R}_{S}\right)}{\alpha kT}\right)-1\right]-\frac{\left(U_{L}+I_{L}*R_{S}\right)}{R_{sh}} \end{array}$$

where ${\;I}_{D\;}$ represents the saturation current under no-light conditions, the series and parallel resistances are denoted by ${\;R}_{S\;}$ and $R_{sh}$, respectively, ${\;U}_{L}$ is the voltage of the PV cell, α is the diode ideality factor, T is the temperature, k denotes the Boltzmann constant, q is the elementary charge, and $I_{ph}$ can be approximately calculated as follows.

(3) $$\begin{array}{c} I_{ph}=\frac{G}{G_{STC}}\left[I_{ph,STC}+\alpha_{sc}\left(T-T_{sc}\right)\right] \end{array}$$

In Formula (3), $G_{STC}$ is the reference irradiance of $1000\;W/m^{2}$, ${\;I}_{ph,STC\;}$ represents the short-circuit current of the solar cell, G is the actual solar irradiance, $T_{sc}$ denotes the reference temperature, and the temperature coefficient of the short-circuit current is denoted by $\alpha_{sc}$.

In practice, since the value of ${\;R}_{sh\;}$ is large and that of ${\;R}_{S}$ is small, the last term in Formula (2) can be neglected, reducing the equation to

(4) $$\begin{array}{c} I_{L}=I_{ph}-I_{D}\times \left[exp\left(\frac{q\left(U_{L}+I_{L}{*R}_{S}\right)}{\alpha kT}\right)-1\right] \end{array}$$

In PSCs, the output current can be modeled as Formula (5):

(5) $$\begin{array}{c} I_{L}=N_{p}\left\{I_{ph}-I_{D}\times \left[exp\left(\frac{q\left(U_{L}+I_{L}{*R}_{S}\right)}{N_{s}*\alpha kT}\right)-1\right]\right\}-\frac{\left(U_{L}+I_{L}*R_{S}\right)}{R_{sh}} \end{array}$$

where ${\;N}_{p}$ and $N_{s}$ represent the number of PV modules connected in parallel and series, respectively. Table 1 presents the specific parameters of the PV cells.

### 2.2. The Effects of PSCs on PV Cells

In PV power generation systems, PSCs represent a critical challenge constraining energy conversion efficiency and system reliability. Currently, conventional PV array configurations typically employ a combination of series- and parallel-connected PV cell modules. When partial shading causes irradiance imbalance among components, the current in series-connected branches becomes limited by the minimum photogenerated current of the shaded modules. Meanwhile, unshaded modules transition from power-generating units to energy-consuming loads due to reverse bias. Under these circumstances, shaded modules endure reverse voltages and accumulate heat from power dissipation, triggering the hot-spot effect. This phenomenon induces localized temperature surges, accelerates the degradation of encapsulation materials and physical damage to solar cells, and significantly shortens module lifespan [41]. This process not only causes instantaneous power loss but may also lead to irreversible component failure due to accumulated thermal stress, jeopardizing system operational safety. To mitigate hot-spot risks, PV systems widely integrate bypass diodes in parallel with series-connected branches [42]. When reverse voltages emerge in shaded branches, the diodes conduct and bypass faulty modules, thereby limiting power dissipation. However, this protective mechanism exhibits dual limitations: on the one hand, the forward voltage drop introduced during diode conduction reduces the overall system efficiency; on the other hand, the bypass activation alters the electrical topology of the array, resulting in a multimodal nonlinear P-V characteristic curve where GMPP coexists with multiple LMPPs. Conventional MPPT algorithms, based on gradient search principles, frequently converge to suboptimal local peaks in multi-peak scenarios, resulting in persistent energy losses.

## 3. Elk Herd Optimizer Algorithm

The EHO algorithm was proposed in 2024 by Al-Betari et al. [38]. In the EHO algorithm, the competition, cooperation, and evolutionary mechanisms of elk herds during the rutting season, calving season and selection season are simulated based on their natural reproductive behavior.

### 3.1. Initialize the Population of EHO

Initially, the elk herd (EH) is generated, representing the population of elk solutions comprising bulls and harem groups. The EH is structured as a matrix of size n×EHS, as shown in Formula (6).

(6) $$EH=\left[\begin{array}{cc} \begin{array}{cc} x_{1}^{1} & x_{2}^{1}\\ x_{1}^{2} & x_{2}^{2} \end{array} & \begin{array}{cc} \ldots & x_{d}^{1}\\ \ldots & x_{d}^{2} \end{array}\\ \begin{array}{cc} \vdots & \vdots \\ x_{1}^{N} & x_{2}^{N} \end{array} & \begin{array}{cc} \ldots & \vdots \\ \ldots & x_{d}^{N} \end{array} \end{array}\right]$$

where N denotes the population size. The position of each elk is calculated using Formula (7).

(7) $$\begin{array}{c} x_{j}^{i}={lb}_{j}+\left({ub}_{j}-{lb}_{j}\right)\times rand \end{array}$$

where $x_{j}^{i}$ denotes the position of the i elk in the j dimension, ${ub}_{j}$ represents the upper bound of the j dimension, ${lb}_{j}$ indicates the lower bound of the j dimension, and rand is a uniformly distributed random value within the range [0, 1]. The fitness value of each elk’s solution is determined using Formula (6). The dominance of elks in EH is ranked according to their fitness values.

### 3.2. Rutting Season

During the rutting season, the elk herd establishes family groups based on the bull ratio $B_{r}$, where the total number of families is calculated as $B=\left|B_{r}\times N\right|$. The herd selects the bulls according to their fitness values, with the top B individuals possessing the highest fitness values in the EH designated as bulls, as shown in Formula (8).

(8) $$B=arg\underset{j=(1,\;2,\;\ldots ,\;B)}{\min}f(x^{j})$$

The roulette wheel selection method is employed to allocate the harems to each bull in the herd, where the harems are distributed among family groups in proportion to the fitness values of the bulls, as defined in Formula (9).

(9) $$\begin{array}{c} p_{j}=\frac{\left|f\left(x^{i}\right)\right|}{\left|\sum_{k=1}^{B}f\left(x^{k}\right)\right|} \end{array}$$

where $p_{j}$ represents the selection probability for each bull $x^{i}$ in B, $f(x^{k})$ denotes the absolute fitness value of bull $x^{i}$, $\left|\sum_{k=1}^{B}f(x^{k})\right|$ indicates the total absolute fitness value of all bulls, and B is the parameter used to calculate the number of family groups.

### 3.3. Calving Season

In calving season, the calves, $x_{i}^{j}\left(t+1\right),$ in each family group are reproduced primarily based on the attributes of the father bull, $x^{h_{j}}\left(t\right),$ and mother harem, $x_{i}^{j}(t)$. If calf $x_{i}(t+1)$ shares the same index i as its bull father in the family, the calf is reproduced as defined in Formula (10).

(10) $$\begin{array}{c} x_{i}^{j}\left(t+1\right)=x_{i}^{j}\left(t\right)+\alpha \cdot \left(x_{j}^{k}\left(t\right)-x_{i}^{j}\left(t\right)\right) \end{array}$$

where $x_{i}^{j}(t+1)$ represents the i elk in the j dimension during the t+1 iteration, ${\;x}_{j}^{k}(t)$ denotes a randomly selected elk from the herd, where k∈(1,2,…,EHS), and α is a random value within the range [0, 1] that determines the proportion of stochastic elements participating in new calf reproduction. When the calf has the same index as its mother, the calf inherits characteristics from both its father bull and harem mother, as formulated in Formula (11).

(11) $$\begin{array}{c} x_{i}^{j}\left(t+1\right)=x_{i}^{j}\left(t\right)+\beta \left(x_{i}^{h_{j}}\left(t\right)-x_{i}^{j}\left(t\right)\right)+\gamma \left(x_{i}^{r}\left(t\right)-x_{i}^{j}\left(t\right)\right) \end{array}$$

where $x_{i}^{h_{j}}(t)$ is the bull of the harem j, r is the index of a randomly selected bull from the current bull set such that r∈B, γ and β are two random values within the range [0, 2], and the inherited traits from previously generated calves are randomly determined.

### 3.4. Selection Season

In selection season, all family members (including bulls, harems, and calves) are merged into a unified solution space. Specifically, the population matrix, EH, which stores the current generation of bulls and harems, and the new solution matrix, ${EH}^{\prime }$, which stores the calves, are consolidated into a temporary matrix EH_temp. Subsequently, all individuals in EH_temp are sorted in ascending order based on their fitness values, and the top EHS individuals are selected as the next-generation population to replace the original population matrix EH.

## 4. Improved EHO-Based MPPT Method

**Figure 2:**
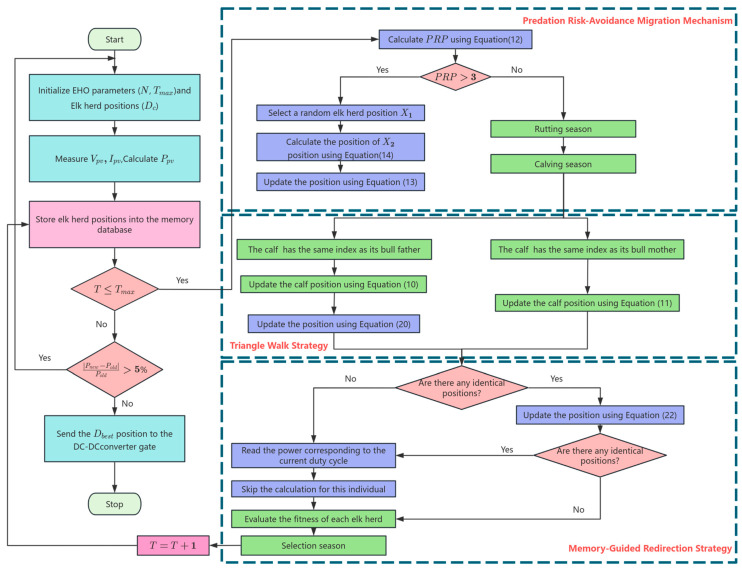
The flowchart of the proposed IEHO MPPT method.

Due to the tendency of EHO to converge to local optima and its slow convergence rate, this paper proposes an improved EHO algorithm for MPPT, aiming to enhance the accuracy of global maximum power tracking and optimize the convergence speed to reduce tracking time. The overall flowchart of the IEHO-MPPT algorithm is illustrated in Figure 2.

### 4.1. Initialize Parameters

In the IEHO-MPPT method, the positions of elk individuals are treated as duty cycles. After each iteration, the algorithm processes voltage and current data from the PV system to compute and optimize the best duty cycle D that satisfies the target power output. For initializing the duty cycle parameters, this study selects six agents with their initial duty cycle positions uniformly distributed within the interval [0, 1]. Given a population size N=6, N+2=8, equally spaced points including the boundaries are generated within the interval [0, 1] through uniform discretization. Subsequently, the first and last boundary points are removed to obtain the initial duty cycle set, $D_{c}$.

### 4.2. Predation Risk Avoidance Migration Mechanism

When facing predation pressure from predators such as wolves and bears, elk herds exhibit migration behaviors toward relatively safer environments. This predator-driven migration constitutes an adaptive strategy rooted in survival–threat responses. At its core, this migration reflects an evolutionarily derived risk threshold response mechanism: when the potential mortality caused by predation pressure exceeds the energetic costs and resource losses associated with migration, the herd prioritizes spatial relocation. By actively disengaging from high-risk environments, elk balance survival and reproductive priorities.

Predation risk probability (PRP) is a dynamic parameter that quantifies the threat posed by predators to elk herds within their current habitat. It combines predator activity intensity, the defensive capabilities of elk, and environmental concealment to simulate how elk herds adjust migration decisions based on real-time risk assessments. The PRP is defined by the following formula:

(12) $$\begin{array}{c} PRP=C*exp\left(-\lambda \cdot N_{p}\times \left(\frac{T}{T_{max}}\right)\right)\times \left(\frac{1}{1+k\cdot N_{e}}\right) \end{array}$$

where $N_{p\;}$ represents the number of active predators within the current habitat, and $N_{e\;}$ is the number of the bulls in the elk herd, determined by the bull ratio, $B_{r}$, which reflects the group’s defensive capacity. λ denotes the predator attack efficiency coefficient, with its value set to 2.6. k signifies the defense efficacy coefficient of the bulls, quantifying the risk reduction capability of individual males, with its value assigned as 0.12. C corresponds to the baseline environmental concealment coefficient, characterizing the attenuation effect of habitat features such as vegetation coverage and terrain complexity on predator attack efficiency, with its value fixed at 6.

In PRP, the number of the bulls is set as $N_{e}=B_{r}\times N$, and this study adopts an active predator count of $N_{p}=randi([1,\;2])$ within the current habitat. When PRP≤3, this indicates minimal predation pressure on the elk herd in the current region, signifying suitability for continued habitation. When PRP>3, the elk herd initiates migration to evade core predator activity zones and seek regions with superior survival conditions. During this phase, the herd’s positional update is calculated by Formula (13).

(13) $$\begin{array}{c} X\left(t+1\right)=X_{2}+\left(X_{1}-X\left(t\right)\right)\times cos\left(\theta \right)\times \left(\frac{1}{PRP}\right)\times H\times rand+X_{1}\times sin\left(\theta \right)\times \left(\frac{1}{PRP}\right)\times H\times rand \end{array}$$

where $X_{1\;}$ is a random position in the current population, $X_{2\;}$ denotes a random position between the candidate optimal position and the current position, which is calculated by Formula (14), and H is the adaptive environmental factor, which is calculated by Formula (15).

(14) $$\begin{array}{c} X_{2}=X\left(t\right)+\left(X_{best}-X\left(t\right)\right)\times rand \end{array}$$

(15) $$\begin{array}{c} H=u\times \left[1-\cos\left(\frac{\pi }{2}\times \left(1-\frac{T}{T_{max}}\right)\right)\right] \end{array}$$

where u is the environmental factor constant with its value set to 1.2. In the predation risk avoidance migration mechanism, θ is defined as a random angle between 0 and 360 degrees, which signifies that the elk herd migrates in a stochastic direction. The randomness in the herd’s positional updates enhances its global search capability, thereby preventing the population from converging to local optima.

### 4.3. Triangle Walk Strategy

The triangle walk strategy [43] enables populations to escape local optima and expand the search scope during positional updates by integrating stochastic step sizes, directional selection, and geometric computations. Therefore, this study employs the triangle walk strategy to further enhance the global search capability of elk herds. First, each elk calculates the distance between its current position and the candidate optimal position, denoted as $L_{1}$, as shown in Formula (16). Subsequently, the elk randomly generates a step size range $L_{2\;}$ and defines the walking direction by selecting a random angle, β. ${\;L}_{2}$ and β are formulated in Formula (17) and Formula (18), respectively. The cosine theorem in Formula (19) is then applied to calculate the distance P between the new position after the walk and the candidate optimal position. The final position update formula for the elk herd is given by Formula (20).

(16) $$\begin{array}{c} L_{1}={pos}_{best}\left(t\right)-pos\left(t\right) \end{array}$$

(17) $$\begin{array}{c} L_{2}=rand\times L_{1} \end{array}$$

(18) $$\begin{array}{c} \beta =2\times \pi \times rand \end{array}$$

(19) $$\begin{array}{c} P=L_{1}^{2}-L_{2}^{2}-2\times L_{1}\times L_{2}\times cos\left(\beta \right) \end{array}$$

(20) $$\begin{array}{c} {Pos}_{new}=pos\left(t\right)+r_{g}\times P \end{array}$$

where $r_{g\;}$ is a dynamic parameter that decreases with the iteration count, serving to balance the intensity of exploration and exploitation. As shown in Formula (21), the value of S is set to 2.

(21) $$\begin{array}{c} r_{g}=S\times exp\left[-{\left(\frac{S\times T}{T_{max}}\right)}^{2}\right] \end{array}$$

### 4.4. Memory-Guided Redirection Strategy

The memory-guided redirection strategy is an enhanced approach that optimizes search trajectories using historical information. Its core principle involves constructing a dynamic memory archive to store historical positions and corresponding fitness values of population individuals within the search space. When the algorithm detects duplicated positions between newly generated individuals and existing entries in the memory archive, it directly retrieves historical fitness values to bypass redundant calculations. Simultaneously, the strategy dynamically adjusts search directions based on the global optimal solution recorded in the memory archive and regenerates unexplored positions. By proactively avoiding duplicated search regions and reusing historical computational results, this strategy significantly reduces algorithmic computational overhead. Meanwhile, it utilizes memorized information to steer the population away from local oscillations or inefficient search paths, thereby achieving accelerated convergence rates and enhanced global search efficiency in complex optimization problems.

#### 4.4.1. Memory Archive Construction

We initialize a storage space to record the position coordinates of population individuals and their corresponding fitness values, structured in the format $X_{i}:f(X_{i})$, where ${\;X}_{i}$ represents the position vector and $f(X_{i})$ denotes the fitness value. M serves as the index for current population positions, and $M_{max\;}$ indicates the maximum capacity of the storage space. When $\left|M\right|\leq M_{max}$, the positions of population M are directly stored. When $\left|M\right|>M_{max}$, M resets to its initial value of 1, and the replacement logic is executed: the memory archive is sorted based on fitness values, and new population positions progressively replace individuals with inferior fitness values in the archive. The memory archive is constructed with a maximum storage capacity of $M_{max}=15\%\cdot T_{max}\times num$.

#### 4.4.2. Memory Detection

During each iteration, when an individual generates a new position $X_{new}$, the memory detection mechanism is activated to query whether duplicate position $X_{new\;}$ exists in the memory archive. If no duplicate is detected, conventional fitness evaluation is performed, and the memory archive is updated. If a duplicate position is identified, the individual employs the dynamic path regeneration mechanism to regenerate a new position, $X_{new}^{\prime }$. The memory detection mechanism is then reapplied to determine the existence of duplicate position $X_{new}^{\prime }$ in the memory archive. If a duplicate is found, the system directly retrieves $f(X_{new}^{\prime })$ and skips the fitness computation step. If no duplicate exists, conventional fitness evaluation is executed, and the memory archive is updated accordingly.

Due to limitations in the system’s floating point precision, exact matches of duty cycle values cannot be detected. The experimental observations reveal that system power remains nearly constant for duty cycles with minor differences. To address this, this study employs a dynamic threshold to determine whether there exists an identical duty cycle in the memory archive. During the exploration phase, the threshold of 0.03 is adopted, while during the exploitation phase, the threshold is set to 0.0001. In the global exploration phase, the algorithm requires the rapid traversal of potential solution spaces to avoid local optima. This necessitates larger duty cycle adjustment steps and a higher threshold to accelerate convergence. Conversely, in the local exploitation phase, the algorithm focuses on fine-grained searches within the neighborhood of optimal solutions, requiring smaller adjustment steps and a reduced threshold to enhance precision. By dynamically modulating the sensitivity of duty cycle matching, the algorithm indirectly controls its search granularity: coarse-grained exploration for accelerated convergence initially, followed by fine-grained exploitation for improved accuracy.

#### 4.4.3. Dynamic Path Regeneration Mechanism

Memory-guided regeneration: When a duplicate position is detected, the individual generates a perturbation direction based on the global optimal position in the memory archive and its current position, calculated using Formula (22).

(22) $$\begin{array}{c} X_{new}^{\prime }={Memory}_{max}+\left({Memory}_{max}-X_{new}\right)\times rand \end{array}$$

where $X_{new\;}$ denotes the individual’s current position, and ${Memory}_{max\;}$ denotes the global optimal position in the memory archive.

### 4.5. Environmental Change

When environmental conditions undergo abrupt changes, the maximum output power and optimal duty cycle of the PV system are altered. Consequently, the algorithm must detect weather variations to restart the MPPT process. The rate of change in the PV output power is measured and computed. If the absolute value of the ratio of the power difference between a given moment and the previous moment to the power at that moment exceeds 5%, a restart mechanism is triggered, which requires the following condition to be met:

(23) $$\begin{array}{c} \frac{\left|P_{new}-P_{old}\right|}{P_{old}}>5\% \end{array}$$

### 4.6. Computation Complexity of IEHO

The computational complexity of the IEHO algorithm is primarily determined by two phases: population initialization and the iterative optimization process. Let N be the population size, $T_{max\;}$ be the maximum number of iterations, and Dim be the problem dimension (where Dim=1 for MPPT), and the computational complexity of the initialization phase is O(N×Dim). The iterative optimization phase includes the foundational EHO update mechanism and three improvement strategies: the predation risk avoidance migration mechanism, the triangle walk strategy, and the memory-guided redirection strategy. The predation risk avoidance migration mechanism and the triangle walk strategy primarily involve position calculations for each individual, and the computational complexity of each strategy is $O({\;T}_{max\;}\times N\times Dim)$. The memory-guided redirection strategy aims to reduce redundant computations, and its maximum computational complexity can be represented as $O({\;T}_{max\;}\times N\times Dim)$.

Therefore, the total computational complexity of the IEHO algorithm can be expressed as $O({\;(4T}_{max\;}+1)\times N\times Dim)$.

## 5. Results and Discussions

**Figure 3:**
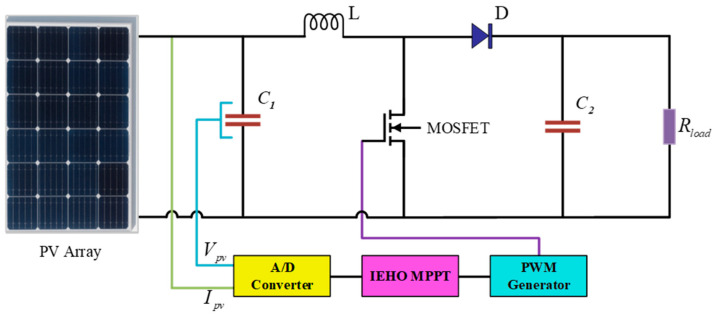
PV system with IEHO MPPT control.

**Figure 4:**
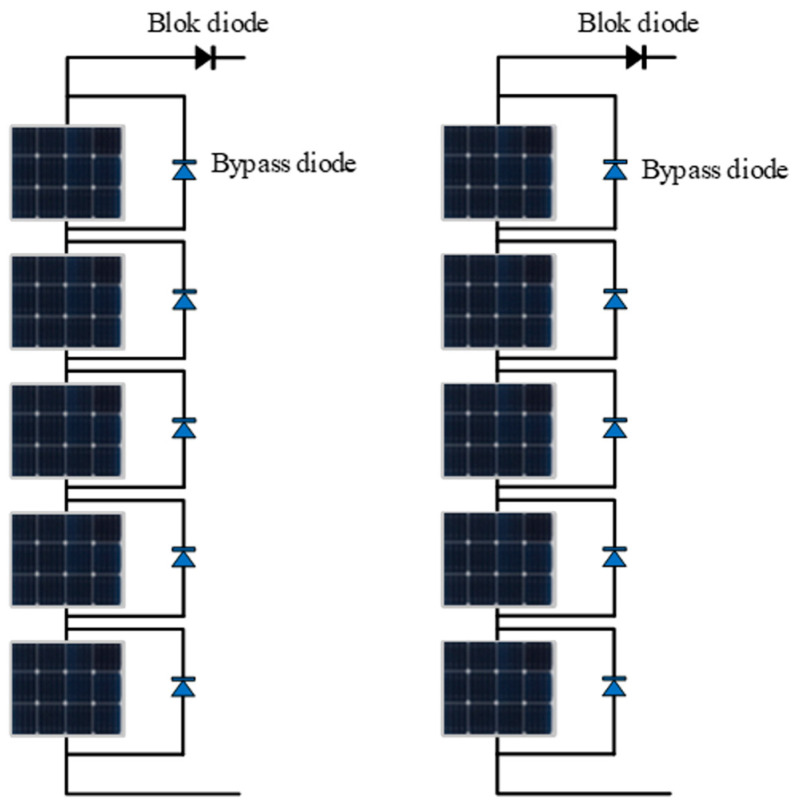
PV module structure with a 5S2P configuration.

**Figure A1:**
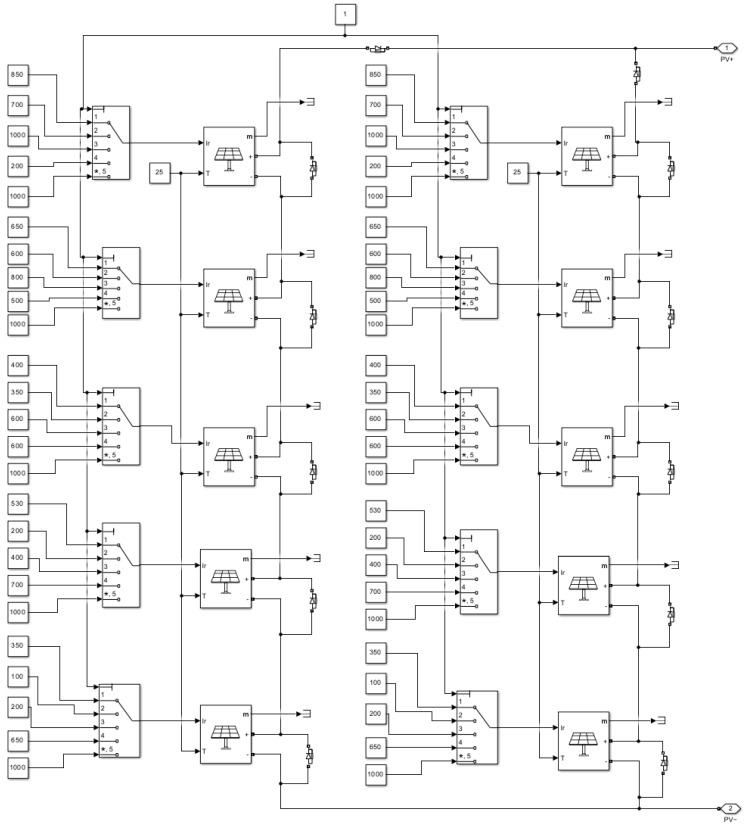
Simulink model of the PV Array.

In this study, the PV MPPT control system primarily comprises series–parallel PV module configurations, a DC-DC boost converter, an IEHO-MPPT controller, and load, as shown in Figure 3, where the circuit component parameters are as follows: $C_{1}=500\;\mu F$, $C_{2}=20\;\mu F$, L=8500 μH, $R_{load}=40\;\Omega .$ The PV array is configured in a 5S2P PV module connection, which consists of two parallel strings with five series-connected modules per string, as illustrated in Figure 4. Figure A1 shows the Simulink model of the PV array developed for this study. The proposed methodology was systematically validated through model construction on the MATLAB/Simulink R2024a simulation platform, implemented on a personal computing platform equipped with a 2.80 GHz Intel(R) Core(TM) i9 CPU with 32 GB RAM. This validation rigorously evaluates the power generation performance of the MPPT algorithm under uniform irradiation, partial shading, and dynamic irradiation conditions.

To evaluate the effectiveness of the proposed method under varying weather conditions, the IEHO-MPPT controller is compared with six other metaheuristic MPPT controllers, including PSO, GWO, SCSO, the SSA, RIME and EHO. Experimental setups and operational conditions remain consistent across all algorithms. The control parameters used for each algorithm are listed in Table 2. The source codes for the IEHO algorithm and the six other comparative algorithms are available at https://github.com/Wei-866/IEHO-MPPT-code, accessed on 10 August 2025. The performance of the algorithm in this study is evaluated using the following metrics.

1. Tracking efficiency

Tracking efficiency is defined as the ratio of the maximum steady-state power attained by the MPPT algorithm to the theoretical maximum power, calculated as follows:

(24) $$\begin{array}{c} \eta_{MPPT}=\frac{{MPPT}_{steady}}{{MPPT}_{available}}\times 100\% \end{array}$$

2. Tracking time

Tracking time refers to the time required for the tracking algorithm to reach steady-state power or achieve convergence.

3. Relative error (RE) and root mean square error (RMSE)

RE and RMSE quantitatively evaluate the steady-state performance of the algorithm after the maximum power point is found. A lower value for RE indicates that the optimization deviation of the algorithm is small, while a lower RMSE value comprehensively reflects the algorithm’s performance, demonstrating both high accuracy and high stability. They are calculated as follows:

(25) $$\begin{array}{c} RE=\left|\frac{P_{avg}-P_{mpp}}{P_{mpp}}\right|\times 100\% \end{array}$$

(26) $$\begin{array}{c} RMSE=\sqrt{\frac{\sum_{i=1}^{n}{\left(P_{i,steady}-P_{mpp}\right)}^{2}}{n}} \end{array}$$

where ${\;P}_{avg}$ is the average output power of the algorithm over the steady-state interval, ${\;P}_{mpp}$ represents the theoretical maximum power point under the current irradiance and temperature conditions, and $P_{i,steady}$ denotes the actual output power during the  i-th run.

### 5.1. Algorithm Performance Analysis Under Uniform Irradiance Conditions

**Figure 5:**
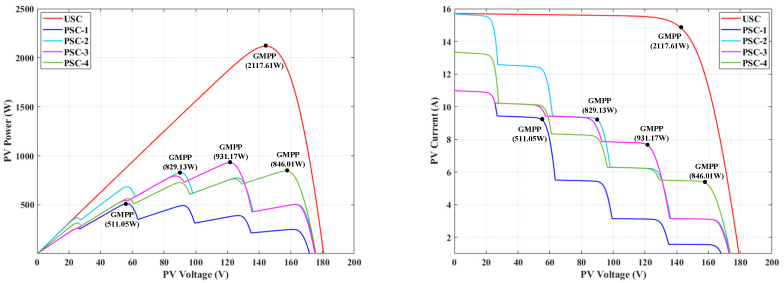
PV and IV characteristic curves of uniform and 4 partial shaded patterns.

**Figure 6:**
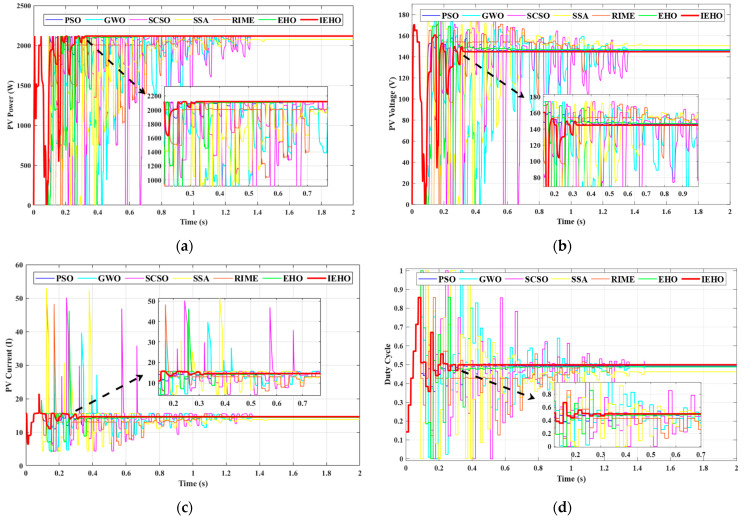
(**a**) PV power, (**b**) PV voltage, (**c**) PV current and (**d**) duty cycle waveform curves of PSO, GWO, SCSO, SSA, RIME, EHO and IEHO algorithms concerning USC.

To validate the effectiveness of the proposed algorithm compared to other algorithms under the USC, the 5S2P-configured PV panel was exposed to an irradiance of 1000 $W/m^{2}$ and an ambient temperature of 25 °C. The PV array exhibited a unimodal power characteristic profile with a single GMPP, as shown in Figure 5, where the maximum peak power reached 2117.61 W. Figure 6 presents the simulation waveforms of the proposed IEHO algorithm and six other algorithms regarding PV power, PV voltage, PV current, and duty cycle under the USC. Table 3 summarizes the simulation results of these algorithms under the USC.

In the USC, the proposed IEHO algorithm achieved the highest steady-state output power of 2117.30 W, with an MPPT efficiency of 99.99%. This was followed by RIME, SCSO, GWO and PSO, which attained steady-state output powers of 2117.21 W, 2116.93 W, 2115.82 W and 2114.82 W, respectively, with MPPT efficiencies of 99.98%, 99.97%, 99.92% and 99.97%. The EHO algorithm achieved a steady-state output power of 2111.41 W and the tracking efficiency of 99.71%. The SSA exhibited the poorest performance, with a tracking efficiency of only 98.05% and a steady-state output power of 2076.25 W.

The experimental results demonstrate that the proposed IEHO algorithm exhibits significant advantages in MPPT speed. Under identical simulation conditions, the IEHO algorithm successfully tracked the maximum power within only 0.3397 s, with the duty cycle parameter stably converging to 0.499. In comparison, the tracking times for PSO, GWO, SCSO, the SSA, RIME and EHO were 0.6727 s, 1.4606 s, 1.3735 s, 1.4607 s, 1.2692 s and 0.8275 s, respectively. The tracking time of IEHO is 49.5% shorter than that of the best-performing comparator algorithm, PSO. Notably, all comparator algorithms exhibited substantial power oscillations during maximum power tracking, leading to additional power losses. In contrast, the IEHO algorithm showed no significant power oscillations when tracking the GMPP. This improvement stems from the predation risk avoidance migration mechanism and memory-guided redirection strategy embedded in IEHO, which effectively address the tendency of traditional swarm intelligence algorithms to become trapped in local oscillations within flat regions of the power curve. These results validate the advantages of IEHO in rapid response and strong robustness.

### 5.2. Algorithm Performance Analysis Under PSCs

To validate the algorithm’s performance under PSCs, this study constructed four complex partial shading scenarios with multi-peak characteristics. As shown in Figure 5, each scenario corresponds to distinct irradiance combinations, and their associated P-V and I-V characteristic curves exhibit significant multi-peak features, each containing four LMPPs and one GMPP. This design aims to simulate real-world power curve distortions caused by cloud cover, building shadows, and similar obstructions in PV systems, focusing on evaluating the algorithm’s capability in extrema identification and the avoidance of local oscillations within multi-peak regions.

**Figure 7:**
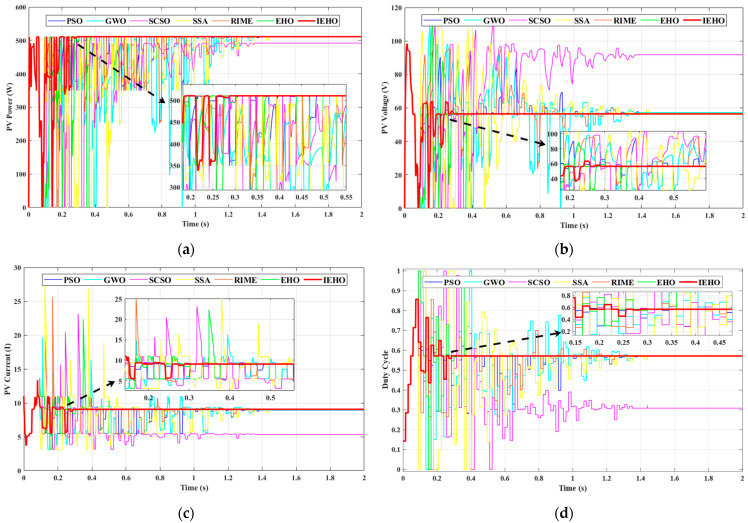
(**a**) PV power, (**b**) PV voltage, (**c**) PV current and (**d**) duty cycle waveform curves of PSO, GWO, SCSO, SSA, RIME, EHO and IEHO algorithms concerning PSC-1.

In PSC-1, the 5S2P-configured PV panel was subjected to irradiance levels of 700, 600, 350, 200 and 100 $W/m^{2}$, as shown in Figure 5. The PV array under PSC-1 exhibited a GMPP located on the left side of the PV characteristic curve, with a maximum peak power of approximately 511.05 W. Figure 7 illustrates the simulation waveforms of the proposed IEHO algorithm and six other algorithms regarding PV power, PV voltage, PV current, and duty cycle under PSC-1. Table 3 summarizes the simulation results of these algorithms under PSC-1. The simulation results demonstrate that the IEHO algorithm successfully tracked the MPP of 510.99 W within the shortest time of 0.3056 s, achieving an MPPT efficiency of 99.99%. The duty cycle stably converged to 0.571 with no observable power oscillations. The PSO algorithm also tracked the MPP of 510.99 W with 99.99% tracking efficiency; however, its tracking time of 1.3961 s was prolonged by 78.1% compared to IEHO, accompanied by persistent power oscillations near the MPP. The EHO, SSA and RIME algorithms achieved steady-state powers of 510.97 W with 99.98% tracking efficiency, and their tracking times were 0.3871 s, 1.4621 s and 1.2619 s, respectively. Although the EHO algorithm exhibited the fastest convergence speed among the comparator algorithms, it still lagged 21.1% behind IEHO. The GWO algorithm tracked a steady-state power of 510.38 W within 1.3784 s, yielding a tracking efficiency of 99.87%. The SCSO algorithm became trapped in a local optimum, attaining a steady-state power of only 491.87 W within 1.3807 s, corresponding to a tracking efficiency of 96.25%.

**Figure 8:**
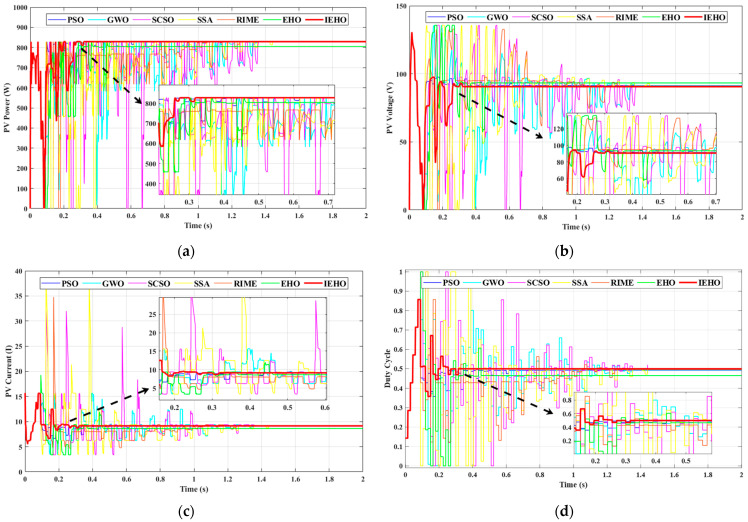
(**a**) PV power, (**b**) PV voltage, (**c**) PV current and (**d**) duty cycle waveform curves of PSO, GWO, SCSO, SSA, RIME, EHO and IEHO algorithms concerning PSC-2.

In PSC-2, the irradiance levels for the 5S2P-configured PV modules are set to 1000, 800, 600, 400 and 200 $W/m^{2}$. As shown in Figure 5, under PSC-2, the GMPP of the PV array is located near the midpoint of the PV characteristic curve, with a maximum peak power of approximately 829.13 W. Figure 8 illustrates the simulation waveforms of the proposed IEHO algorithm and six other algorithms regarding PV power, PV voltage, PV current, and duty cycle under PSC-2. Table 3 summarizes the simulation results of the algorithms under PSC-2. The simulation results indicate that the IEHO algorithm achieved the shortest convergence time, with a tracking duration of 0.3297 s, a tracked maximum power of 828.97 W, an MPPT efficiency of 99.98%, and a duty cycle stably converging to 0.499. Notably, no significant power oscillations were observed in IEHO after reaching the MPP. Among the comparator algorithms, PSO, GWO, RIME, the SSA and SCSO attained steady-state powers of 827.24 W, 827.79 W, 828.35 W, 828.56 W and 828.74 W, respectively, with tracking efficiencies of 99.77%, 99.84%, 99.91%, 99.93% and 99.95%, and tracking times of 0.6700 s, 1.3700 s, 1.2726 s, 1.4648 s and 1.3781 s. Although the PSO algorithm demonstrated the fastest convergence (0.6700 s) among the comparator algorithms, it still lagged 50.8% behind IEHO and exhibited intermittent power fluctuations. The EHO algorithm became trapped in a local optimum, yielding a steady-state power of 805.28 W and a tracking efficiency of 97.12%, which were significantly inferior to other algorithms. Despite its convergence time of 0.5035 s outperforming some comparator algorithms, EHO remained 34.5% slower than IEHO.

**Figure 9:**
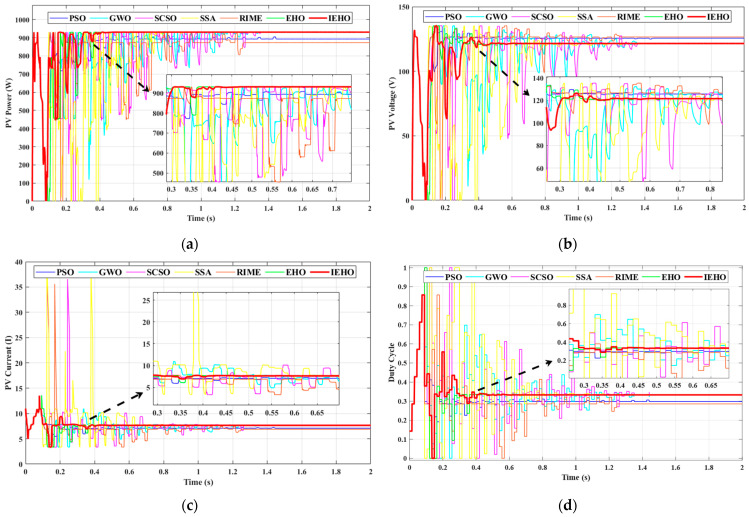
(**a**) PV power, (**b**) PV voltage, (**c**) PV current and (**d**) duty cycle waveform curves of PSO, GWO, SCSO, SSA, RIME, EHO and IEHO algorithms concerning PSC-3.

In PSC-3, the irradiance levels for the 5S2P-configured PV modules are set to 200, 500, 600, 700 and 650 $W/m^{2}$. As shown in Figure 5, under PSC-3, the GMPP of the PV array is located on the mid-right side of the PV characteristic curve, with a maximum peak power of approximately 931.17 W. Figure 9 illustrates the simulation waveforms of PV power, PV voltage, PV current, and duty cycle for the proposed IEHO algorithm and six comparator algorithms under PSC-3. Table 3 summarizes the simulation results of the algorithms under PSC-3. The simulation results reveal that the proposed IEHO algorithm exhibits remarkable tracking performance. It rapidly converges to the GMPP of 930.99 W within 0.5536 s, achieving an MPPT efficiency of 99.98%. The duty cycle parameter stabilizes at 0.333 with no significant power oscillations, demonstrating exceptional steady-state performance. Among the comparator algorithms, EHO, the SSA and GWO achieve comparable tracking efficiencies of 99.98% and successfully track a steady-state power of 930.98 W. However, their convergence times are extended to 0.6994 s, 1.2726 s and 1.3721 s, respectively, accompanied by notable power fluctuations. Notably, IEHO improves convergence speed by 20.8% and 56.5% compared to EHO and the SSA, respectively, validating its effectiveness in multi-peak scenarios. The SCSO algorithm tracks a steady-state power of 930.96 W within 1.3867 s, with a tracking efficiency of 99.98%. In contrast, PSO and RIME suffer from severe power losses due to convergence to local optima, yielding reduced steady-state powers of 892.90 W and 872.84 W, with tracking efficiencies of 95.89% and 93.74%, respectively. Their convergence times are 1.4721 s and 1.2794 s, respectively. Although RIME converges slightly faster than PSO, it exhibits larger power oscillations.

**Figure 10:**
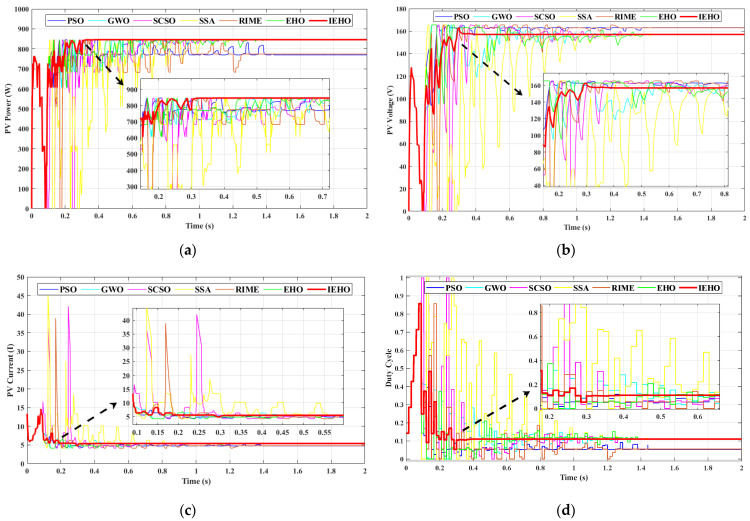
(**a**) PV power, (**b**) PV voltage, (**c**) PV current and (**d**) duty cycle waveform curves of PSO, GWO, SCSO, SSA, RIME, EHO and IEHO algorithms concerning PSC-4.

In PSC-4, the irradiance levels for the 5S2P-configured PV modules are set to 850, 650, 400, 530 and 350 $W/m^{2}$. As shown in Figure 5, under PSC-4, the GMPP of the PV array is located on the right side of the PV characteristic curve, with a maximum peak power of approximately 846.01 W. Figure 10 illustrates the simulation waveforms of PV power, PV voltage, PV current, and duty cycle for the proposed IEHO algorithm and six comparator algorithms under PSC-4. Table 3 summarizes the simulation results of the algorithms under PSC-4. The simulation results demonstrate that, compared to other algorithms, the IEHO algorithm successfully tracks the MPP (845.99 W) in the shortest time (0.4142 s), achieving an MPPT efficiency of 99.99%. The duty cycle stabilizes at 0.111 with no significant power oscillations. GWO and the SSA also attain the MPP of 845.99 W with 99.99% tracking efficiency, but their tracking times are prolonged to 1.3682 s and 1.4065 s, respectively. Further analysis reveals that the SSA exhibits higher power oscillations than GWO, indicating deficiencies in its local search stability. Meanwhile, the EHO and SCSO algorithms exhibit comparable performance, achieving tracking efficiencies of 99.99% with peak powers of 845.97 W and 845.96 W, respectively, and tracking times of 1.4512 s and 1.4011 s. Both algorithms display noticeable power oscillations. PSO and RIME perform the worst, with tracking efficiencies of 90.97% and 91.48%, tracked powers of 769.64 W and 773.99 W, and convergence times of 1.4733 s and 1.2843 s, respectively. Both exhibit persistent power oscillations, indicating their susceptibility to local optima in multi-peak scenarios. Notably, the tracking time of IEHO is reduced by 69.7% compared to the best-performing comparator algorithm (GWO), confirming the proposed algorithm’s significant advantage in tracking speed.

An analysis of the experimental results in Table 3 demonstrates that among all compared algorithms, the proposed IEHO algorithm exhibits the most significant advantages in both RE and RMSE metrics. Specifically, under all tested conditions, IEHO achieves an average RE of only 0.0156%. This result indicates that IEHO delivers the highest steady-state tracking accuracy, maintaining the photovoltaic system’s operating point closest to the theoretical maximum power point over extended periods, thereby ensuring exceptional energy harvesting efficiency. Meanwhile, IEHO yields the lowest average RMSE among all algorithms at merely 0.2299. As a comprehensive metric, this low RMSE value not only reconfirms IEHO’s high accuracy but, more critically, reflects its minimal steady-state oscillation in output power. In summary, the outstanding results in both RE and RMSE collectively verify the superior steady-state performance of the IEHO algorithm, which combines exceptionally high optimization precision with excellent output stability.

### 5.3. Algorithm Performance Analysis Under Dynamic Irradiance Changes

In practical PV power generation scenarios, the irradiance received by PV arrays often exhibits dynamic, non-uniform, and complex characteristics due to factors such as cloud movement, shifting obstructions, and fluctuating ambient light intensity. These variable energy inputs frequently induce multi-peak phenomena and sudden jumps in the system’s power output curve, imposing stringent demands on the real-time performance and stability of MPPT algorithms. Therefore, this section validates the tracking performance of the IEHO algorithm under dynamic irradiance scenarios through combined dynamic irradiance experiments.

**Figure 11:**
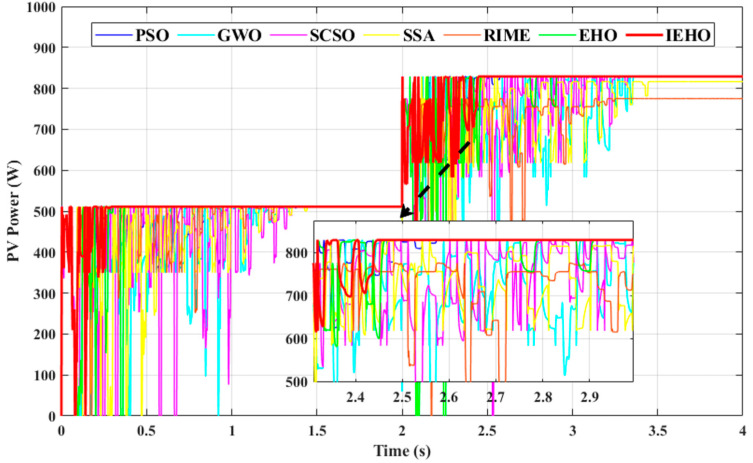
PV power waveform curves of PSO, GWO, SCSO, SSA, RIME, EHO and IEHO algorithms under the transition from PSC-1 to PSC-2 conditions.

The simulation experiment constructs irradiance conditions in two phases: the PV system operates under PSC-1 irradiance from 0 to 2 s; at t = 2 s, the system irradiance undergoes a stepwise abrupt change and switches to PSC-2 irradiance. The simulation waveforms of PV power for the proposed IEHO algorithm and six comparator algorithms under dynamic irradiance conditions are shown in Figure 11. The results demonstrate that when illumination shifts to PSC-2, the IEHO algorithm successfully tracks the MPP (828.97 W) in the shortest time (2.4650 s), achieving a tracking efficiency of 99.98%. The steady-state powers tracked by PSO, GWO, SCSO and EHO are 828.91 W, 828.05 W, 828.64 W and 828.88 W, respectively, with tracking efficiencies of 99.97%, 99.87%, 99.94% and 99.97% and tracking times of 2.7158 s, 3.3924 s, 3.3896 s and 2.9359 s. The SSA and RIME fail to track the MPP, yielding steady-state powers of 816.44 W and 774.97 W, tracking efficiencies of 98.47% and 93.47%, and tracking times of 3.4727 s and 3.2770 s. Table 4 summarizes the simulation results for the RE and RMSE of the algorithms under dynamic irradiance conditions. The experimental results demonstrate that the proposed IEHO algorithm possesses outstanding steady-state performance. In summary, the IEHO algorithm rapidly reinitializes power searches in dynamic environments and exhibits superior tracking performance compared to other algorithms. These findings further validate IEHO’s exceptional adaptability and robustness under dynamic irradiance conditions.

### 5.4. Summary of Experimental Analysis

**Figure 12:**
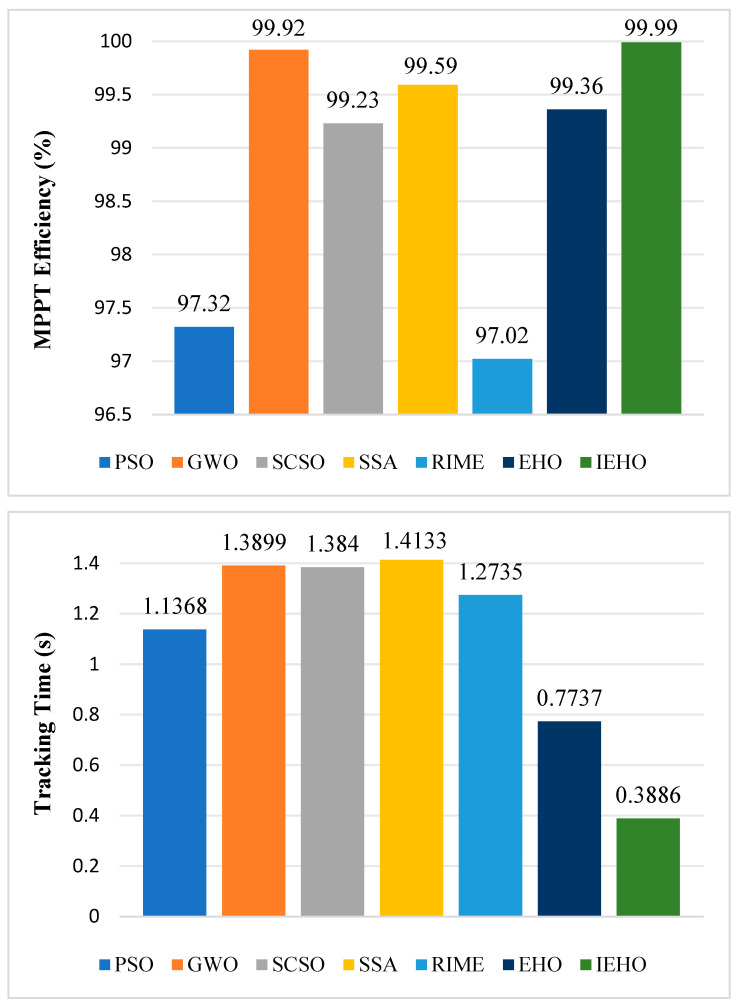
The average tracking time and efficiency under the USC and PSCs.

The experimental results demonstrate that the proposed IEHO algorithm outperforms other comparative methods in both tracking speed and accuracy under both the USC and PSCs. Figure 12 presents a comparison of the average tracking times and average MPPT efficiencies of the IEHO algorithm and six other algorithms across all-weather conditions. As shown in Figure 12, the IEHO algorithm achieves superior tracking accuracy and shorter tracking times compared to all other algorithms under all tested conditions. IEHO attains the fastest average tracking time of 0.3886 s and the highest average efficiency of 99.99%. In contrast, RIME and PSO exhibit the poorest tracking performance, frequently converging to local optima, with average tracking efficiencies of only 97.02% and 97.32%, respectively. Additionally, both algorithms suffer from significant power oscillations after reaching steady-state power. Although GWO, SCSO and the SSA achieve relatively high average tracking efficiencies, their average tracking times are prolonged by 1.0013 s, 0.9954 s and 1.0247 s, respectively, compared to IEHO. Notably, even the EHO algorithm, which achieves the fastest tracking performance among all comparator algorithms, demonstrates a 0.3851 s longer average tracking time and a 0.63% lower average tracking efficiency compared to the IEHO algorithm. The experimental data conclusively demonstrate that, across complex multi-scenario tests, the IEHO algorithm significantly outperforms other comparative algorithms in core performance metrics, including convergence speed, tracking accuracy, and anti-interference capability.

## 6. Conclusions

MPPT technology enables PV systems to operate at the MPP, thereby enhancing PV cell utilization. However, PV systems under PSCs exhibit multi-peak P-V characteristics, which often cause conventional MPPT algorithms to converge to local optima, reducing energy conversion efficiency and system stability. To address these challenges, this study proposes an IEHO algorithm that incorporates three mechanisms: the predation risk avoidance migration mechanism, triangle walk strategy and memory-guided redirection strategy. The predation risk avoidance migration mechanism enables elk herds to perceive and evade high-risk zones while stochastically selecting secure habitat spaces for position updates, thereby enhancing global search capabilities. The triangle walk strategy expands the search scope to strengthen escape from local optima, while the memory-guided redirection strategy accelerates convergence by eliminating redundant recalculations of duplicate duty cycles.

In this study, the proposed algorithm was compared with other MPPT techniques under various irradiance conditions, including PSO, GWO, SCSO, the SSA, RIME and EHO. The results indicate that the proposed IEHO algorithm outperforms existing MPPT techniques in both tracking time and tracking efficiency, achieving an average tracking efficiency of 99.99% and an average tracking time of 0.3886 s across all-weather conditions. Furthermore, the algorithm significantly mitigates power oscillations near the GMPP, ensuring stable operation under diverse environmental conditions and effectively improving the power generation efficiency of PV systems. Future research could explore hybrid optimization strategies that integrate the IEHO algorithm with conventional methods, other optimization algorithms or machine learning techniques for PV system MPPT applications, further enhancing PV conversion efficiency. Additionally, the proposed algorithm could be applied to both off-grid and grid-connected PV systems to validate its practical applicability in real-world scenarios.

## Figures and Tables

**Table 1:** Parameter specifications of PV Cell.

Parameter	Values
Open-circuit voltage ($U_{oc}$)	36.3 V
Short-circuit current ($I_{sc}$)	7.84 A
Voltage at maximum power point ($U_{m}$)	29 V
Current at maximum power point ($I_{m}$)	7.35 A
Temperature coefficient of $U_{oc}$ (α)	−0.36099%/℃
Temperature coefficient of $I_{sc}$ (β)	0.102%/℃

**Table 2:** Tuning parameters of PSO, GWO, SCSO, SSA and RIME.

Metaheuristic Methods	Parameter
PSO	$C_{1}=1.2$$,{\;C}_{2}=1.2$, w=0.1.
GWO	a=decreases from 2 to 0.
SCSO	$S_{M}=2$, θ=[0,360].
SSA	C1=2×exp(−(4×TTmax)^2)$,{\;C}_{2},C_{3}=rand(0,1)$.
RIME	w=5.

**Table 3:** The simulation results of the algorithm under USC and 4 PSCs.

Metrics	Scenario	PSO	GWO	SCSO	SSA	RIME	EHO	IEHO
Tracking Time (s)	USC	0.6727	1.4606	1.3735	1.4607	1.2692	0.8275	0.3397
	PSC-1	1.3961	1.3784	1.3807	1.4621	1.2619	0.3871	0.3056
	PSC-2	0.67	1.37	1.3781	1.4648	1.2726	0.5035	0.3297
	PSC-3	1.4721	1.3721	1.3867	1.2726	1.2794	0.6994	0.5536
	PSC-4	1.4733	1.3682	1.4011	1.4065	1.2843	1.4512	0.4142
	Average	1.1368	1.3899	1.384	1.4133	1.2735	0.7737	0.3886
MPPT Efficiency (%)	USC	99.97	99.92	99.97	98.05	99.98	99.71	99.99
	PSC-1	99.99	99.87	96.25	99.98	99.98	99.98	99.99
	PSC-2	99.77	99.94	99.95	99.93	99.91	97.12	99.98
	PSC-3	95.89	99.98	99.98	99.98	93.74	99.98	99.98
	PSC-4	90.97	99.99	99.99	99.99	91.48	99.99	99.99
	Average	97.32	99.92	99.23	99.59	97.02	99.36	99.99
RE (%)	USC	0.1415	0.0964	0.0322	2.0441	3.5221	0.3244	0.0147
	PSC-1	1.0006	0.1319	3.9819	1.4389	2.6183	1.1276	0.0123
	PSC-2	0.2464	0.1624	0.0476	0.9776	3.7381	2.877	0.0191
	PSC-3	4.0145	0.0399	0.2972	0.0568	8.115	0.1575	0.0269
	PSC-4	8.3867	0.0777	0.2707	0.1167	9.0149	1.6356	0.0051
	Average	2.7579	0.1016	0.9259	0.9268	5.4016	1.2244	0.0156
RMSE	USC	3.3762	2.1373	0.6832	43.8643	182.6571	7.1364	0.3145
	PSC-1	22.8662	0.6767	20.894	25.5048	41.848	55.51	0.0684
	PSC-2	2.3538	1.3474	0.3973	27.1226	55.1274	23.8544	0.1608
	PSC-3	37.5609	0.9191	10.8543	1.0828	104.3368	12.3215	0.4299
	PSC-4	72.1824	3.1856	6.014	3.9101	83.0098	30.0113	0.176
	Average	27.6679	1.6532	7.7685	20.2969	93.3958	25.7667	0.2299

**Table 4:** Simulation results of the algorithm under dynamic irradiance changes.

Metrics	Scenario	PSO	GWO	SCSO	SSA	RIME	EHO	IEHO
RE (%)	0–2 (s)	1.0007	0.1319	0.0257	1.4389	2.6181	1.1278	0.0123
	2–4 (s)	0.0306	0.1299	0.0614	2.6713	6.7496	0.3082	0.0195
RMSE	0–2 (s)	22.8663	0.6767	0.1379	25.5049	41.8440	55.5131	0.0684
	2–4 (s)	0.2879	1.0790	0.5202	37.5014	56.2020	12.0403	0.1666

## Data Availability

The data presented in this study are available on request from the corresponding author.

## Abbreviations

The following abbreviations are used in this manuscript:

AI	Artificial intelligence
ANN	Artificial neural network
BWO	Beluga whale optimization
CNN	Convolutional neural network
CS	Cuckoo search
CVT	Constant voltage tracking
DBO	Dung beetle optimization
DE	Differential evolution
EHO	Elk herd optimization
FLC	Fuzzy logic control
GA	Genetic algorithm
GMPP	Global maximum power point
GWO	Grey wolf optimization
HC	Hill climbing
HHO	Harris hawk optimization
HHO	Horse herd optimization
IEHO	Improved elk herd optimization
INC	Incremental conductance
ISO	Improved snake optimizer
LMPPs	Local maximum power points
MPPT	Maximum power point tracking
P&O	Perturbation and observation
PRP	Predation risk probability
PSCs	Partial shading conditions
PSO	Particle swarm optimization
PV	Photovoltaic
P-V	Power–voltage
RE	Relative error
RMSE	Root mean square error
SCSO	Sand cat swarm optimization
SSA	Salp swarm algorithm
USC	Uniform irradiance condition
ZOA	Zebra optimization algorithm
